# Infant formulas with synthetic oligosaccharides and respective marketing practices: Position Statement of the German Society for Child and Adolescent Medicine e.V. (DGKJ), Commission for Nutrition

**DOI:** 10.1186/s40348-022-00146-y

**Published:** 2022-07-13

**Authors:** Christoph Bührer, Regina Ensenauer, Frank Jochum, Hermann Kalhoff, Berthold Koletzko, Burkhard Lawrenz, Walter Mihatsch, Carsten Posovszky, Silvia Rudloff

**Affiliations:** 1 Klinik für NeonatologieCharité — Universitätsmedizin Berlin, Berlin, Germany; 2 Institut für Kinderernährung, Max-Rubner-Institut, Karlsruhe, Germany; 3 Evangelisches Waldkrankenhaus Berlin-Spandau, Berlin, Germany; 4 Klinik für Kinder- und Jugendmedizin, Klinikum Dortmund, Dortmund, Germany; 5 Kinderklinik Und Kinderpoliklinik, Dept. of Pediatrics, Dr. von Hauner Children’s Hospital, University Hospital, LMU Munich, Munich, Germany; 6 Praxis für Kinder- und Jugendmedizin, Arnsberg, Germany; 7 Fakultät Gesundheitsmanagement, Hochschule Neu-Ulm, Neu-Ulm, Germany; 8 Universitäts-Kinderspital Zürich, Zürich, Switzerland; 9 Institut für Ernährungswissenschaft, Justus-Liebig-Universität Giessen, Giessen, Germany

**Keywords:** Breastfeeding, Infant formula, Food additives, Marketing of breast milk substitutes, Health claims

## Abstract

Human milk contains more than 150 different oligosaccharides, which together are among to the quantitatively predominant solid components of breast milk. The oligosaccharide content and composition of human milk show large inter-individual differences. Oligosaccharide content is mostly influenced by genetic variants of the mother’s secretor status. Oligosaccharides in human milk are utilized by infants’ intestinal bacteria, affecting bacterial composition and metabolic activity. Maternal secretor status, and respective differing fucosylated oligosaccharide content, has been associated both with reduced and increased risk of infection in different populations of breastfed infants, possibly due to environmental conditions and the infant’s genotype. There are no safety concerns regarding the addition of previously approved oligosaccharides to infant formula; however, no firm conclusions can be drawn about clinically relevant benefits either. Therefore, infant formulas with synthetic oligosaccharide additives are currently not preferentially recommended over infant formulas without such additives. We consider the use of terms such as “human milk oligosaccharides” and corresponding abbreviations such as “HMO” in any advertising of infant formula to be an inappropriate idealization of infant formula. Manufacturers should stop this practice, and such marketing practices should be prevented by responsible supervisory authorities. Pediatricians should inform families that infant formulas supplemented with synthetic oligosaccharides do not resemble the complex oligosaccharide composition of human milk.

**Supplementary Information:**

The online version contains supplementary material available at 10.1186/s40348-022-00146-y.

## Background

Human milk contains lactose as a digestible carbohydrate and various oligosaccharides as indigestible carbohydrates. In mature human milk, the total content of oligosaccharides is 5–15 g/L. Together with lactose, fat, and protein, they are one of the major solid components of human milk [1, 2]. Oligosaccharides in human milk (known as “breast milk oligosaccharides,” “human milk oligosaccharides,” or “HMOs”) are made up of five building blocks, namely galactose, glucose, fucose, N-acetyl glucosamine, and N-acetyl neuraminic acid [3]. Beginning with lactose, the complexity of the diverse structures increases through one or multiple extensions with lacto-N-biose or lactosamine and additional modifications with fucose and/or sialic acid. The activity of various short- and long-chain components of mammalian milk has been characterized [4]. Of these, about two-thirds is neutral, and one-third is acidic (containing sialic acid) oligosaccharides. There are 15 predominant oligosaccharides that account for 80–90% of the total content of oligosaccharides in human milk.

## Individual variations and genetic predisposition

The oligosaccharide patterns in human milk show very large inter-individual differences which are partly genetically determined. In humans, certain clusters can be distinguished by the presence or absence of certain glycosyltransferases, such as fucosyltransferases FUT2 and FUT3 [5, 6]. FUT2 mediates the synthesis of the neutral oligosaccharides such as 2′-FL and lacto-N-fucopentaose-I (LNFP-I). FUT3 is crucial for the formation of lacto-N-fucopentaose-II (LNFP-II). FUT2-positive mothers have higher concentrations of oligosaccharides in their milk than FUT2-negative mothers [7]. Mothers who express FUT2 are referred to as “secretors” because α1-2-fucosylated oligosaccharides are detectable in their milk. In contrast, such components are absent in nonsecretors (inactive FUT2 gene). In Europe, about 70–80% of the population are secretors, and 20–30% are nonsecretors [8].

The biological significance of the differences in human milk composition between secretors and nonsecretors (of 2′-FL) is a matter of debate. Lack of FUT2 activity has been associated with relative resistance to rotavirus and norovirus infections [9–11] but increased colonization rate with group B streptococci [12]. Divergent effects have been reported in different populations. Studies from North America showed a lower incidence of diarrhea in breastfed children of secretors than in breastfed children of nonsecretors [13, 14], while breastfed children of secretors in the UK, Bangladesh, Peru, and Tanzania showed increased diarrhea incidence [15, 16]. An association of the level of 2′-FL in milk with excessive weight gain in infants has also been reported [17]. The effects of secretor status may differ depending on environmental conditions and pathogen exposure. In addition to the composition of human milk, the infant’s secretor status also seems to be important. Infant FUT2 and FUT3 positivity were associated with a marked risk reduction by almost 30% for all-cause diarrhea [15]. However, further data from clinical studies are required to potentially support conclusive inferences.

## Biological functions of oligosaccharides in human milk

Oligosaccharides pass undigested through the small intestine but are metabolized by gut bacteria. They can affect metabolic activity and proliferation of the intestinal microbiota, similar to the effects of undigested lactose and fiber. With regard to the structural variety and the sometimes very high content of certain oligosaccharides in human milk, structure-specific effects have also been ascribed to them [1, 2, 18, 19]. An ever-increasing number of ex vivo and animal studies indicates potential gastrointestinal and systemic effects. Effects on the composition of the intestinal microbiome have been the most studied so far. Oligosaccharides conveyed through human milk seem to be preferentially metabolized by certain commensal bacteria, in particular *Bifidobacteria* and *Bacteroides* species. Bacteria that utilize certain oligosaccharides (“cross-feeding”) and receptor-analogous effects of oligosaccharides may influence intestinal colonization and the composition of the microbiota (through, for example, the formation of short-chain fatty acids). An infant’s immune system could be influenced directly or indirectly via the composition of the microbiota. Furthermore, certain oligosaccharides interfere with the lectin-mediated binding of certain pathogenic bacteria or viruses to the intestinal mucosa [12, 20]. Influences on intestinal permeability and intestinal cell maturation are also debated [1, 21].

The total amount of oligosaccharides in milk does not differ between mothers of preterm infants with and without necrotizing enterocolitis (NEC) [22, 23]. However, human milk fed to preterm infants who developed NEC had less disialyllacto-N-tetraose (DSLNT) than milk fed to control infants in studies conducted in South Africa [24], North America [22], and the UK [25], whereas NEC was associated with less milk lacto-N-difucohexaose I and lower diversity of oligosaccharides in a Swedish cohort [23]. In randomized controlled trials, pasteurized human milk has been shown to reduce the risk of NEC in preterm infants [26]. It is conceivable that human milk oligosaccharides which are not affected by pasteurization might contribute to the observed risk reduction for NEC.

Since small amounts of oligosaccharides can be taken up systemically, leukocyte-endothelium interactions detected in vitro or the effect on lymphocytes with the subsequent production of specific cytokines is also conceivable in vivo [27]. There is also some evidence to suggest that oligosaccharides may influence the gut-brain axis. In rodents and pigs, the use of oligosaccharides had a positive effect on the development of brain functions [28, 29]. However, it is currently unclear whether these experimental animal data reflect the situation in human infants.

## Oligosaccharides in cow’s milk and goat’s milk

In cow’s milk, which serves as the basis for the production of infant formula, there are only a few mainly acidic oligosaccharides, present in very low concentrations. The total content in mature cow’s milk is about 0.03–0.06 g/L. In goat’s milk, which is also used for producing infant formula [30], concentrations of 0.06–0.35 g/L are slightly higher than in cow’s milk [31].

## Addition of synthetic oligosaccharides to infant formula

Oligosaccharides have been added to some infant formulas. Galactooligosaccharides (GOS) are galactose oligomers synthesized from lactose. GOS including 3′-galactosyllactose (3′-GL) are found in human milk only in small amounts [32–34]. Fructooligosaccharides (FOS), also called oligofructose, are fructose polymers which have a sweetening effect. They are absent in human milk. In clinical studies, the addition of short-chain GOS and long-chain FOS in a ratio of 9:1 [30, 35], which is approved in Europe, at a concentration of 0.8 g/100 mL led to softer stool consistency and an increase in the proportion of *bifidobacteria* in infants’ stool [36]. No conclusive data are available for any other effect [36]. The European Food Safety Authority (EFSA) did not find evidence for any cause-effect relationship between the intake of GOS or FOS and reductions in gastrointestinal discomfort or potentially pathogenic microorganisms [37, 38].

Advances in the production of oligosaccharides, including the use of genetically modified microorganisms, have made it possible to produce some of the oligosaccharides found in human milk on an industrial scale [21, 39, 40]. However, only simple, short-chain oligosaccharides are currently used, mostly because of financial costs. EFSA and US Food and Drug Administration (FDA) have evaluated several synthetic oligosaccharides also found in human milk as novel food ingredients (2′-fucosyllactose, 2′-FL; lacto-N-neotetraose, LNnT; lacto-N-tetraose, LNT; 2′-FL + difucosyllactose, DFL; 3′-sialyllactose, 3′-SL; and 6′-sialyllactose, 6′-SL) [41–46]. Table 1 shows the maximum levels of synthetic oligosaccharides or combinations of oligosaccharides permitted for addition to infant formulas.

**Table 1:** The European Food Safety Authority (EFSA) maximum permitted levels of structurally identical synthetic human milk oligosaccharides or oligosaccharide combinations in infant formulas (in g/L)

**Oligosaccharide**	Infant formulas	Infant follow-on formulas
2′-FL	2.4	2.4
2′-FL + DFL	1.6	1.2
LNnT	0.6	0.6
LNT	0.8	0.6
3′-SL	0.2	0.15
6′-SL	0.4	0.3

## Recent clinical studies on infant formula supplemented with synthetic oligosaccharides

At present, there are only a few clinical studies in which the supplementation of infant formula with 2′-FL alone or in combination with LNnT or other nondairy oligosaccharides (GOS) has been investigated [27, 47–49].

Marriage et al. reported in 2015 that the supplementation of infant formula with 2′-FL (control 2.4 g GOS; experimental infant formula 1: 2.2 g GOS + 0.2 g 2′-FL; experimental infant formula 2: 1.4 g GOS + 1.0 g/l 2′-FL) did not lead to significant differences in head circumference, height or weight of the infants in the experimental groups, compared to breastfed infants, over the first 4 months. In addition, the authors state that the supplemented formula was well tolerated, and that the amount of 2′-FL detected in blood was comparable to that in breastfed infants [47].

Two randomized studies with infant formulas to which 2′-FL [50] or 2′-FL and LNnT [48] had been added showed no adverse effects on infant growth or tolerance to the formula. As a secondary endpoint, fewer respiratory infections and less use of antipyretics and antibiotics in the first year of life were reported when using infant formula enriched with 2′-FL and LnNT compared to non-supplemented formula [48]. These findings require further verification. In a further clinical study on an infant formula supplemented with different concentrations of GOS, with or without the addition of 2′-FL, the authors describe a lower inflammatory cytokine profile in the first 4 months of life that is comparable to that of exclusively breastfed children [27]. The addition of 2′-FL + LNnT to infant formula has also been reported to affect bacterial populations in infants’ stool [49].

In summary, no disadvantages in terms of infant growth have been observed in infants fed infant formulas supplemented with individual oligosaccharides previously approved by EFSA. Reported effects on the infant’s gut microbiota and the defense against infections require confirmation in further studies. As reported above, some oligosaccharides such as 2′-FL are absent from human milk in 20–30% of mothers in Europe. Both advantages and disadvantages with regard to risk of infections in breastfed infants of nonsecretory mothers have been described in different studies. It is unknown whether the addition of fucosylated oligosaccharides to infant formula could analogously induce both potential benefits and risks. However, the existence of individual oligosaccharides in human milk alone is not a sufficient justification for an assumed additional benefit of structurally identical synthetic oligosaccharides in infant formula. The oligosaccharide fraction in human milk is highly complex and has an individualized composition. Whether these differences affect the health of the infant cannot be assessed at this time. Moreover, the complexity of the oligosaccharides in human milk currently cannot be emulated in infant formula [51]. Overall, existing data on supplementation of infant formula with synthetic oligosaccharides are considered too limited to make general recommendations for its use.

## Marketing of infant formulas fortified with synthetic oligosaccharides

In their marketing to consumers, manufacturers of infant formulas and follow-on formulas enriched with synthetic oligosaccharides suggest a similarity with breastfeeding. They do this by using terms such as “breast milk oligosaccharides” or “human milk oligosaccharides” (“HMO”) on product packaging, on their websites, through sponsored blogs, and in magazine articles. The use of this term suggests to consumers that the oligosaccharide composition in infant formula is similar to that of human milk. This is not correct and can lead to consumer deception, because the addition of simple, short-chain oligosaccharides does not lead to a similarity with the complex composition of hundreds of short- and long-chain oligosaccharides in human milk.

The Committee on Nutrition regards this kind of marketing as a violation of applicable European and German law. The European Union directive on infant formula and follow-on formula states that communication on infant formula “should not undermine the promotion of breastfeeding.” Furthermore, “use of the terms ‘humanised,’ ‘maternalised,’ ‘adapted,’ or similar terms is prohibited” [52]. The German regulation of dietetic foods prohibits “idealized wording” in the labelling of infant formula. Accordingly, when labelling infant formula and follow-on formula, the use of the terms “humanized,” “maternalised,” “adapted,” or similar terms is prohibited [53]. The Commission for Nutrition considers terms such as “breast milk oligosaccharides” or “human milk oligosaccharides” and respective abbreviations such as “HMO” in relation to infant formula to be misleading. Idealization of infant formula with the term “humanized” and similar terms is considered to be equally unlawful and undermine the promotion of breastfeeding.

## Additional information

The German version of this consensus article can be found as an additional file attached to this article.

## Conclusions

- The Commission for Nutrition of the German Society for Child and Adolescent Medicine does not see any safety concerns when supplementing infant formulas with the synthetic oligosaccharides previously approved in Europe in the specified maximum amounts.
- The few studies on infants available to date do not allow any reliable conclusions to be drawn about clinically relevant advantages of synthetic oligosaccharide additives.
- Preferential use of infant formulas with synthetic oligosaccharide additives is therefore not recommended on the basis of currently available data.
- The use of terms such as “human milk oligosaccharides” and abbreviations such as “HMO” in promoting infant and follow-on formula represents an unacceptable idealization, which suggests a nonexistent similarity with human milk and can thus undermine the priority of breastfeeding promotion.
- The Committee on Nutrition urges infant formula manufacturers to end the current unacceptable idealized promotion of infant formula. It calls on supervisory authorities to stop possible violations of the existing legal restrictions on the marketing of infant formula.
- Pediatricians should inform families that synthetic oligosaccharides in infant formula do not match the complex composition of oligosaccharides found in human milk.

## Supplementary Information

**Additional file 1.** The German version of the article.
