# Correction: Infant formulas with synthetic oligosaccharides and respective marketing practices: Position Statement of the German Society for Child and Adolescent Medicine e.V. (DGKJ), Commission for Nutrition

**DOI:** 10.1186/s40348-022-00147-x

**Published:** 2022-07-23

**Authors:** Christoph Bührer, Regina Ensenauer, Frank Jochum, Hermann Kalhoff, Berthold Koletzko, Burkhard Lawrenz, Walter Mihatsch, Carsten Posovszky, Silvia Rudloff

**Affiliations:** 1 Klinik für NeonatologieCharité — Universitätsmedizin Berlin, Berlin, Germany; 2 Institut für Kinderernährung, Max-Rubner-Institut, Karlsruhe, Germany; 3 Evangelisches Waldkrankenhaus Berlin-Spandau, Berlin, Germany; 4 Klinik für Kinderund Jugendmedizin, Klinikum Dortmund, Dortmund, Germany; 5 Kinderklinik Und Kinderpoliklinik, Department of Pediatrics, Dr. von Hauner Children’s Hospital, University Hospital, LMU Munich, Munich, Germany; 6 Praxis für Kinder- und Jugendmedizin, Arnsberg, Germany; 7 Fakultät Gesundheitsmanagement, Hochschule Neu-Ulm, Neu-Ulm, Germany; 8 Universitäts-Kinderspital Zürich, Zürich, Switzerland; 9 Institut für Ernährungswissenschaft, Justus-Liebig-Universität Giessen, Giessen, Germany

**Correction: Mol Cell Pediatr 9, 14 (2022)**

**https://doi.org/10.1186/s40348-022-00146-y**

Following publication of the original article [1], authors would like to correct the article title. The correct article title is given below:

Infant formulas with synthetic oligosaccharides and respective marketing practices: Position Statement of the German Society for Child and Adolescent Medicine e.V. (DGKJ), Commission for Nutrition.

The correction does not have any effect on the results or conclusions of the paper. The original article has been corrected.
