# Supplementary material for: Infant formulas with synthetic oligosaccharides and respective marketing practices: Position Statement of the German Society for Child and Adolescent Medicine e.V. (DGKJ), Commission for Nutrition
Source: Mol Cell Pediatr. 2022 Jul 13;9:14. doi: 10.1186/s40348-022-00146-y (PMC9279532; doi:10.1186/s40348-022-00146-y)
Supplement: Supplementary file 1 — Additional file 1. The German version of the article. [file 40348_2022_146_MOESM1_ESM.pdf]

Zusatz synthetischer Oligosaccharide zu Säuglingsnahrungen und deren Bewerbung

Stellungnahme der Ernährungskommission der Deutschen Gesellschaft für Kinder- und Jugendmedizin e.V. (DGKJ): Christoph Bührer[1], Regina Ensenauer[2], Frank Jochum[3], Hermann Kalhoff[4], Berthold Koletzko (Vorsitzender)[5], Burkhard Lawrenz[6], Walter Mihatsch[7], Carsten Posovszky[8], Silvia Rudloff[9]

[1]Klinik für Neonatologie, Charité – Universitätsmedizin Berlin, [2]Institut für Kinderernährung, Max-Rubner-Institut, Karlsruhe, [3]Evangelisches Waldkrankenhaus Berlin Spandau, [4]Klinik für Kinder- und Jugendmedizin, Klinikum Dortmund, [5]LMU – Ludwig Maximilians Universität München, Kinderklinik und Kinderpoliklinik, Dr. von Haunersches Kinderspital, LMU Klinikum München, [6]Praxis für Kinder- und Jugendmedizin, Arnsberg, [7]Fakultät Gesundheitsmanagement, Hochschule Neu-Ulm, [8]Universitäts-Kinderspital Zürich, [9]Institut für Ernährungswissenschaft, Justus-Liebig-Universität Gießen

Korrespondenz:

Prof. Dr. Dr. h.c. mult. Berthold Koletzko, c/o DGKJ e.V., Chausseestr. 128/129, 10115 Berlin

Zusammenfassung:

Menschliche Milch enthält mehr als 150 unterschiedliche Oligosaccharide, die insgesamt zu den quantitativ überwiegenden Feststoffkomponenten der Muttermilch gehören. Die Oligosaccharidgehalte und -zusammensetzung der Muttermilch zeigen große inter-individuelle Unterschiede, die u.a. durch genetische Varianten des Sekretorstatus bedingt sind. Oligosaccharide der Milch werden von kindlichen Darmbakterien verwertet und wirken sich auf deren Zusammensetzung und Stoffwechselaktivität aus. Der mütterliche Sekretorstatus und damit die unterschiedlichen Milchgehalte an fukolysierten Oligosacchariden wurden in unterschiedlicher Population bei den gestillten Kindern sowohl mit reduzierten als auch mit erhöhten Infektionsrisiken assoziiert, möglicherweise abhängig von Umgebungsbedingungen und kindlichem Genotyp. Für die Zugabe bisher zugelassener Oligosaccharide zu Säuglingsnahrungen bestehen keine

Sicherheitsbedenken, aber es lassen sich auch keine belastbaren Schlussfolgerungen auf klinisch relevante Vorteile ziehen. Deshalb wird eine bevorzugte Verwendung von Säuglingsnahrungen mit synthetischen Oligosaccharid-Zusätzen derzeit nicht empfohlen. Wir schätzen die Verwendung von Begriffen wie *„Humane Milch-Oligosaccharide“* und entsprechenden Abkürzungen wie *„HMO“* bei der Bewerbung von Säuglingsnahrung als unzulässige Idealisierung ein, die von Herstellern beendet und von den zuständigen Überwachungsbehörden unterbunden werden soll. Kinder- und Jugendärzt*innen sollten Familien informieren, dass die praktizierte Anreicherung von Säuglingsnahrungen mit synthetischen Oligosacchariden keine Ähnlichkeit mit der komplexen Oligosaccharid-Zusammensetzung der Muttermilch erreicht.

Schlüsselwörter:

Stillen, Säuglingsnahrung, Lebensmittelzusatzstoffe, Vermarktung von Muttermilchersatzprodukten, Gesundheitsbezogene Angaben

Abstract:

Human milk contains more than 150 different oligosaccharides, which in total belong to the quantitatively predominant solid components of breast milk. Oligosaccharide contents and composition of human milk show very large inter-individual differences, partly due to genetic variants of the secretor status. Milk oligosaccharides are utilized by infantile intestinal bacteria and affect their composition and metabolic activity. Maternal secretor status and thus different milk levels of fucosylsated oligosaccharides have been associated with both reduced as well as increased infection risks in different populations of breastfed infants, possibly depending on environmental conditions and infant genotype. There are no safety concerns for the addition of currently approved oligosaccharides to infant formulas, but no robust conclusions be drawn regarding clinically relevant benefits. Therefore, the preferential use of infant formulas with added synthetic oligosaccharide is not recommended at this time. We consider the use of terms such as "*human milk oligosaccharides*" and corresponding abbreviations such as "*HMO*" in the promotion of infant formula as an unacceptable idealization, which should be discontinued by manufacturers and be eliminated by the responsible monitoring authorities. Paediatricians should inform families that the currently practiced fortification of infant formulas with synthetic oligosaccharides do not resemble the complex oligosaccharide composition of breast milk.

Key Words:

Breastfeeding, Infant formula, Food ingredients, Marketing of breast milk substitutes, Health claims

Hintergrund

Milch enthält als verdauliches Kohlenhydrat Lactose sowie als unverdauliche Kohlenhydrate verschiedene Oligosaccharide. In reifer menschlicher Milch liegt der Gesamtgehalt der Oligosaccharide bei 5-15 g/l, sie gehören damit zusammen mit Lactose, Fett und Protein mengenmäßig zu den überwiegenden Feststoffkomponenten der Muttermilch [1, 2]. Die in menschlicher Milch enthaltenen Oligosaccharide (sog. Muttermilch-Oligosaccharide oder Human Milk Oligosaccharides, HMO) sind aus den fünf Bausteinen Galactose, Glucose, Fucose, N-Acetyl-Glucosamin und einer spezifischen Sialinsäure (N-Acetyl-Neuraminsäure) aufgebaut [3]. Ausgehend von Lactose steigt die Komplexität der vielfältigen Strukturen durch eine oder mehrfache Verlängerungen mit Lacto-N-Biose oder Lactosamin sowie zusätzlichen Modifizierungen mit Fucose und/oder Sialinsäure an. Durch die Aktivität verschiedener Glycosyltransferasen entstehen fucosylierte (z.B. 2‘-Fucosyllactose, 2‘-FL), neutrale (z.B. Lacto-N-Tetraose (LNT) und Lacto-N-neoTetraose (LNnT)) oder saure Oligosaccharide (z.B. 3‘- und 6’Sialyllactose (3’SL, 6’SL)) [1, 4]. Bisher sind etwa 160 kurz- und langkettige Komponenten aus Säugetiermilchen strukturell aufgeklärt [5]; davon sind etwa 2/3 neutrale und 1/3 saure (Sialinsäure-haltige) Oligosaccharide. Etwa 15 Oligosaccharide machen etwa 80-90% des Gesamtgehalts an Oligosacchariden in Frauenmilch aus.

Individuelle Schwankungen und genetische Prädisposition

Die Oligosaccharidmuster in Frauenmilch zeigen inter-individuell sehr große Unterschiede, die u.a. genetisch bedingt sind. In Abhängigkeit vom Vorhandensein bzw. Nichtvorhandensein bestimmter Glycosyltransferasen lassen sich beim Menschen bestimmte Cluster unterscheiden, die wesentlich durch genetische Variation der Aktivität der Fucosyltransferasen FUT2 und FUT3 bestimmt sind [4, 6]. FUT2 vermittelt die Synthese der neutralen Oligosaccharide wie 2’FL und Lacto-N-fucopentaose-I (LNFP-I), FUT3 ist entscheidend für die Entstehung von Lacto-N-fucopentaose-II (LNFP-II). FUT2-positive Mütter haben höhere Oligosaccharidkonzentrationen in ihrer Milch als FUT2-negative [7]. Mütter, die FUT2 exprimieren, werden als Sekretorinnen bezeichnet, da in ihrer Milch α1-2-fucosylierte Oligosaccharide nachweisbar sind. Bei Nicht-Sekretorinnen (inaktives FUT2-Gen) dagegen fehlen solche Komponenten. In Europa gehören etwa 70-80 % der Bevölkerung zu den Sekretorinnen und 20-30 % zu den Nicht-Sekretorinnen [8]. Die biologische Bedeutung der Unterschiede in der Milchzusammensetzung von Sekretorinnen und Nicht-Sekretorinnen (z.B. 2’FL) wird diskutiert. Fehlende FUT2-Aktivität wurde mit einer relativen Resistenz gegenüber Rota- und Norovirusinfektionen [9–11], aber einer erhöhten Besiedlungsrate mit Gruppe B-Streptokokken assoziiert [12]. Es wurden divergierende Effekte in unterschiedlichen Populationen berichtet. Studien aus Nordamerika zeigten bei gestillten Kindern von Sekretorinnen eine geringere Durchfallhäufigkeit

als bei gestillten Kindern von Nicht-Sekretorinnen [13, 14], während gestillte Kinder von Sekretorinnen in Großbritannien, Bangladesch, Peru und Tansania eine erhöhte Durchfallhäufigkeit zeigten [15, 16]. Möglicherweise unterscheiden sich die Auswirkungen des Sekretorstatus je nach den Umgebungsbedingungen und der Pathogen-Exposition. Des Weiteren scheint neben der Muttermilchzusammensetzung auch der kindliche Sekretorstatus von Bedeutung zu sein: bei FUT2 und FUT3 positiven Kindern wurde ein um fast 30% reduziertes Durchfallrisiko berichtet [15]. Für eine abschließende Beurteilung sind weitere Daten aus klinischen Studien erforderlich.

Biologische Funktionen von Oligosacchariden in Frauenmilch

Da Oligosaccharide unverdaut den Dünndarm passieren aber durch Darmbakterien metabolisiert werden, können sie – ebenso wie unverdaute Lactose und Ballaststoffe – die metabolische Aktivität und die Vermehrung des intestinalen Mikrobioms beeinflussen. Im Hinblick auf die strukturelle Vielfalt und den teilweise sehr hohen Gehalt bestimmter Oligosaccharide in Humanmilch wurden diesen auch strukturspezifische Wirkungen zugeschrieben [1, 2, 17, 18]. Eine mittlerweile fast unüberschaubare Anzahl an *ex vivo* und tierexperimentellen Studien deutet auf mögliche gastrointestinale wie auch systemische Wirkungen hin. Bisher am meisten untersucht sind Wirkungen auf die Zusammensetzung des intestinalen Mikrobioms. Die mit Muttermilch zugeführten Oligosaccharide werden offenbar präferentiell von bestimmten kommensalen Bakterien, insbesondere Bifidobakterien und Bacteroides species, metabolisiert. Durch eine wechselseitige Beeinflussung von Bakterien, die bestimmte Oligosaccharide verwerten („cross-feeding") sowie durch rezeptoranaloge Wirkungen von Oligosacchariden auf die Darmbesiedelung könnte das Immunsystem direkt oder indirekt über die Zusammensetzung der kindlichen Mikrobiota und dadurch z.B. über die Bildung von kurzkettigen Fettsäuren beeinflusst werden. Weiterhin interferieren bestimmte Oligosaccharide mit der Lektin-vermittelten Bindung gewisser pathogener Bakterien oder Viren an die Darmmukosa [12, 19]. Diskutiert werden auch Einflüsse auf die Darmpermeabilität und Darmzellreifung [1, 20].

Die Gesamtmenge von Oligosacchariden in der Milch unterscheidet sich nicht zwischen Müttern von Frühgeborenen mit und ohne nekrotisierender Enterokolitis (NEC) [21, 22]. Weiterführende Analysen sind widersprüchlich (bei NEC weniger Disialyllacto-N-tetraose (DSLNT) bei südafrikanischen [23], nordamerikanischen [21] und englischen [24] Frühgeborenen, weniger Lacto-N-difucohexaose I und geringere Diversität in einer schwedischen Kohorte [22]). Das NEC-Risiko Frühgeborener wird in kontrollierten Studien durch pasteurisierte Frauenmilch reduziert [25]. Es ist denkbar, dass zu dieser Risikoreduktion auch die nach Pasteurisierung erhaltenen Oligosaccharide der Milch beitragen könnten.

Da geringe Mengen an Oligosacchariden systemisch aufgenommen werden können, sind *in vitro* nachgewiesene Leukozyten-Endothel-Interaktionen oder die Wirkung auf Lymphozyten mit der nachfolgenden Produktion spezifischer Zytokine grundsätzlich auch *in vivo* denkbar [26]. Außerdem gibt es Hinweise auf eine mögliche Beeinflussung der Darm-Hirn-Achse durch Oligosaccharide. Bei Nagern und Schweinen wirkte sich der Einsatz von Oligosacchariden positiv auf die Entwicklung von Gehirnfunktionen aus [27, 28]. Gegenwärtig ist ungeklärt, ob diese tierexperimentellen Daten die Situation bei Säuglingen reflektieren.

Oligosaccharide in Kuh- und Ziegenmilch

In Kuhmilch, die als Basis in der Produktion von Säuglingsmilchnahrungen dient, kommen nur wenige, vor allem azide Oligosaccharide in sehr niedrigen Konzentrationen vor (Gesamtgehalt in reifer Kuhmilch etwa 0,03-0,06 g/l). In Ziegenmilch, die teilweise ebenfalls zur Herstellung von Säuglingsmilchnahrungen verwendet wird [29], ist die Konzentration mit etwa 0,06-0,35 g/l etwas höher als in Kuhmilch [30].

Zusatz von synthetischen Oligosacchariden zu Säuglingsnahrungen

Seit vielen Jahren werden einigen Säuglingsnahrungen Oligosaccharide zugesetzt, vor allem Galactooligosaccharide (GOS, aus Lactose synthetisierte Galactoseoligomere) mit 3'Galactosyllactose (3'GL), die in menschlicher Milch nur in geringen Mengen gefunden wurden [31–33], und Fructooligosaccharide (FOS, Fructosepolymere, auch als Oligofructose bezeichnet), die deutlich süßend wirken und nicht in menschlicher Milch vorkommen. Der in Europa zugelassene Zusatz von kurzkettigen GOS und langkettigen FOS im Verhältnis 9:1 [29, 34] führte in klinischen Studien bei Säuglingen bei einer Konzentration von 0,8 g/100 ml zu weicherer Stuhlkonsistenz und zu einer Erhöhung des Anteils von Bifidobakterien im Stuhl, während für andere Wirkungen keine konklusive Datenlage vorliegt [35]. Bezüglich einer Minderung des gastro-intestinalen Missempfindens oder der Reduktion von potentiell pathogenen Mikroorganismen konnte die Europäische Behörde für Lebensmittelsicherheit (EFSA) keine Ursachen-Wirkungsbeziehung mit der Aufnahme an GOS bzw. FOS feststellen [36, 37].

Fortschritte in der Herstellung von Oligosacchariden durch biotechnologische Verfahren, u.a. mit Einsatz genmodifizierter Mikroorganismen, haben es ermöglicht, einige in humaner Milch vorkommende Oligosaccharide in industriellem Maßstab zu produzieren [20, 38, 39]. Dabei kommen auch aus Kostengründen derzeit nur vergleichsweise einfach aufgebaute, kurzkettige Oligosaccharide zum Einsatz. Die EFSA und die US-amerikanische Food and Drug Administration (FDA) haben mehrere

synthetische Oligosaccharide, die auch in menschlicher Milch vorkommen, als neuartige Lebensmittelinhaltsstoffe (Novel Food Ingredients) zur Verwendung als unbedenklich bewertet (2’Fucosyllactose, 2’-FL; Lacto-N-neoTetraose, LNnT; Lacto-N-Tetraose, LNT; 2´FL + Difucosyllactose, DFL; 3´Sialyllactose, 3‘SL und 6´Sialyllactose, 6’SL) [40–45]. Die für einen Zusatz zu Säuglingsnahrungen akzeptierten Höchstmengen solcher synthetischen Oligosaccharide bzw. ihrer Kombinationen zeigt Tabelle 1.

Neuere klinischen Studien zu mit synthetischen Oligosacchariden angereicherten Säuglingsmilchnahrungen

Bisher liegen nur wenige klinischen Studien vor, in denen die Supplementierung von Säuglingsmilchen mit 2´FL alleine oder in Kombination mit LNnT sowie auch in Kombination mit anderen, nicht-milchtypischen Oligosacchariden (GOS) untersucht wurden [26, 46–48].

Marriage et al. berichteten 2015, dass die Supplementierung einer Säuglingsnahrung mit 2´FL (Kontrolle 2,4 g GOS; Experimentelle Säuglingsnahrung 1: 2,2 g GOS + 0,2g 2´FL; Experimentelle Säuglingsnahrung 2: 1,4 g GOS + 1,0 g/l 2´FL) in den ersten 4 Monaten nicht zu signifikanten Unterschieden bezüglich Kopfumfang, Größe und Gewicht der Säuglinge im Vergleich zu gestillten Säuglingen führte. Außerdem geben die Autor*innen an, dass die supplementierte Nahrung gut vertragen wurde und die ins Blut aufgenommene Menge von 2´FL mit derjenigen bei gestillten Kindern vergleichbar war [46].

Zwei randomisierte Studien mit Säuglingsnahrungen, denen 2’FL [49] bzw. 2’FL und LNnT [47] zugesetzt worden waren, zeigen keine nachteiligen Wirkungen auf Wachstum und Verträglichkeit der Säuglingsnahrung. Als sekundärer Endpunkt wird bei Verwendung von mit 2’FL und LnNT angereicherter Säuglingsnahrung gegenüber unsupplementierter Säuglingsnahrung über weniger respiratorische Infekte sowie einen geringeren Einsatz von Antipyretika und Antibiotika im ersten Lebensjahr berichtet [47], was einer weiteren Überprüfung bedarf. In einer weiteren klinischen Studie mit einer angereicherten Säuglingsmilchnahrung mit unterschiedlichen Konzentrationen an GOS mit oder ohne Zusatz von 2‘FL beschreiben die Autor*innen ein mit ausschließlich gestillten Kindern vergleichbares niedrigeres inflammatorisches Zytokinprofil in den ersten vier Lebensmonaten [26]. Bei der Zugabe von 2´FL+LNnT zu einer Säuglingsnahrung wurden auch Auswirkungen auf die Bakterienpopulationen im Stuhl der Säuglinge berichtet [48].

Zusammenfassend kann man festhalten, dass für die Anreicherung von Säuglingsnahrungen mit den von der EFSA bisher zugelassenen einzelnen Oligosacchariden hinsichtlich des Wachstums der

Säuglinge keine Nachteile beobachtet wurden. Mögliche Wirkungen auf die intestinale Mikrobiota des Säuglings und die Infektabwehr bedürfen einer Bestätigung in weiteren Studien. Wie oben berichtet, kommen manche Oligosaccharide wie z.B. 2'FL bei 20-30% der Mütter in Europa nicht in ihrer Milch vor. Wie zuvor beschrieben, wurden bei gestillten Säuglingen solcher Nonsekretorinnen-Mütter aus unterschiedlichen Studien sowohl Vor- als auch Nachteile im Hinblick auf das Risiko für Infektionen beschrieben. Es wurde auch eine Assoziation des Gehaltes an 2'-FL in der Milch mit exzessiver Gewichtszunahme bei Säuglingen berichtet [50]. Es ist nicht bekannt, ob die Zugabe fucolysierter Oligosaccharide zu Säuglingsnahrung in analoger Weise sowohl potenzielle Vorteile als auch Risiken induzieren könnte. Jedoch ist das Vorkommen einzelner Oligosaccharide in Humanmilch für sich allein noch keine ausreichende Begründung für einen anzunehmenden Zusatznutzen von strukturidentischen synthetischen Oligosacchariden in Säuglingsmilchnahrungen. Die Oligosaccharidfraktion in Humanmilch ist sehr komplex und individuell zusammengesetzt; ob sich diese Unterschiede auf die Gesundheit des Säuglings auswirken, kann momentan nicht beurteilt werden. Derzeit kann die personalisierte Komplexität der Oligosaccharide in Frauenmilch in Säuglingsnahrungen nicht nachgeahmt werden [51]. Insgesamt wird die Datenlage zur Supplementierung von Säuglingsnahrungen mit synthetischen Oligosacchariden als zu dürftig eingeschätzt, um allgemeine Empfehlungen zur Anwendung auszusprechen.

Vermarktung von mit synthetischen Oligosacchariden angereicherten Säuglingsnahrungen

Hersteller von mit synthetischen Oligosacchariden angereicherten Säuglingsanfangs- und Folgenahrungen suggerieren in der Vermarktung an die Verbraucher über Packungen, Internetseiten, gesponsorte Blogs und Zeitschriftenartikel u.a. eine größere Ähnlichkeit mit Muttermilch durch die Verwendung von Begriffen wie „Muttermilch-Oligosaccharide" oder „Humane Milch-Oligosaccharide" („HMO"). Die Verwendung dieser Begrifflichkeit suggeriert Verbraucher*innen, dass eine Ähnlichkeit der Oligosaccharidzusammensetzung in Säuglingsnahrungen mit denen der menschlichen Milch gegeben sei. Dies ist sachlich falsch und kann zu einer Verbrauchertäuschung führen, denn der Zusatz von einzelnen einfachen und kurzkettigen Oligosacchariden führt nicht zu einer Ähnlichkeit mit der komplexen Zusammensetzung von hunderten kurz- und langkettigen Oligosacchariden in menschlicher Milch.

Die Ernährungskommission bewertet diese Art der Vermarktung als einen Verstoß gegen geltendes europäisches und deutsches Recht. In der Direktive der Europäischen Union zu Säuglingsanfangsnahrung und Folgenahrung wird ausgeführt, dass die Kommunikation zu Säuglingsnahrungen *„der Förderung des Stillens nicht entgegenwirkt"* und weiter *„Die Verwendung der Begriffe „humanisiert", „maternisiert", „adaptiert" oder ähnlicher Begriffe ist untersagt"* [52]. Die

deutsche Verordnung über diätetische Lebensmittel untersagt bei der Kennzeichnung von Säuglingsanfangsnahrung *„idealisierende Wortlaute“*. Entsprechend ist bei der Kennzeichnung von Säuglingsanfangsnahrung und Folgenahrung die Verwendung der *„Begriffe „humanisiert“, „maternisiert“, „adaptiert“ oder gleichsinnige Begriffe“* untersagt [53]. Die Ernährungskommission hält Begriffe wie *„Muttermilch-Oligosaccharide“* oder *„Humane Milch-Oligosaccharide“* und darauf verweisende Abkürzungen wie *„HMO“* im Zusammenhang mit Säuglingsnahrungen für in gleicher Weise irreführend und idealisierend wie den Begriff *„humanisiert“*, und entsprechend in gleicher Weise für rechtswidrig und für geeignet, der Förderung des Stillens entgegenzuwirken.

Schlussfolgerungen

- Die Ernährungskommission der Deutschen Gesellschaft für Kinder- und Jugendmedizin erkennt keine Sicherheitsbedenken bei der Anreicherung von Säuglingsnahrungen mit den in Europa bisher zugelassenen synthetischen Oligosacchariden in den festgelegten Höchstmengen.
- Die bisher vorliegenden, wenigen Studien bei Säuglingen erlauben derzeit keine belastbaren Schlussfolgerungen auf klinisch relevante Vorteile von synthetischen Oligosaccharid-Zusätzen.
- Eine bevorzugte Verwendung von Säuglingsnahrungen mit synthetischen Oligosaccharid-Zusätzen wird deshalb auf der Basis der derzeitigen Datenlage nicht empfohlen.
- Die Verwendung von Begriffen wie *„Humane Milch-Oligosaccharide“* und darauf verweisende Abkürzungen wie *„HMO“* bei der Bewerbung von Säuglingsnahrung stellen eine unzulässige Idealisierung dar, die eine nicht bestehende Ähnlichkeit mit dem Stillen suggerieren und damit der Priorität der Förderung des Stillens entgegenwirken können.
- Die Ernährungskommission fordert die Hersteller von Säuglingsnahrungen dazu auf, die derzeitige unakzeptable idealisierende Bewerbung von Säuglingsnahrungen zu beenden. Sie fordert die Überwachungsbehörden auf, mögliche Verstöße gegen die bestehenden rechtlichen Beschränkungen zur Bewerbung von Säuglingsnahrungen zu unterbinden.
- Kinder- und Jugendärzt*innen sollten Familien informieren, dass die synthetischen Oligosaccharide in angereicherten Säuglingsnahrungen nicht der komplexen Oligosaccharid-Zusammensetzung in der Frauenmilch entsprechen.

Interessenerklärungen:

C. Bührer war als Berater bzw. Gutachter für öffentliche Gerichte tätig. Er war Mitglied eines wissenschaftlichen Beirats bei Fresenius, beim WIdO und beim IQTIG. Für Vortrags- und Schulungstätigkeiten erhielt CB von Chiesi sowie Nestlé ein Honorar. Das BMBF förderte seine Forschungsvorhaben durch Drittmittel.

R. Ensenauer erhält für ihre Tätigkeit für den Thieme-Verlag Pädiatrie Up2Date eine Aufwandsentschädigung. Das BMBF, der Innovationsfonds des G-BA, die DFG sowie die Stiftungen Sternstunden e.V., Willi-Althof-Stiftung, EKFS und Stiftung Kardiovaskuläre Prävention LMU München förderten Forschungsvorhaben von RE durch Drittmittel.

F. Jochum ist Berater/Gutachter für die DKG, den G-BA und verschiedene Hersteller von Säuglingsnahrung, von denen er Zuwendungen erhält. Er arbeitet im Beirat des Nestlé Nutrition Institutes mit. Für Vortragstätigkeiten für die Intensivpflegeausbildung DHZB, die Med. Hochschule Brandenburg und die Univ. Greifswald erhielt er ein Honorar. Auch war er Autor/Koautor für verschiedene Publikationen mit Fokus zur klinischen Ernährung oder Behandlung von pädiatrischen Patienten einschließlich Neugeborenen. Er führte Forschungsvorhaben mit Fokus auf die Ernährung von Neugeborenen durch, für die er ein Honorar und Reisekostenerstattungen erhielt. FJ besitzt zwei Patente betreffend Neugeborene.

H. Kalhoff erhielt für Vortrags- und Schulungstätigkeiten von Duleve Mederau und Stallergenes ein Honorar.

B. Koletzko ist Mitglied der Begutachtungsgruppe Klinische Studien der Deutschen Forschungsgemeinschaft. Er hielt Erstattungen für Fortbildungsvorträge und gutachterliche Tätigkeiten von den Unternehmen Annenberg, Baxter, Bayer, Cogitando, Cheplapharm, Danone, DSM, Hipp, Nestle, und Reckitt und der gemeinnützigen Familie Larsson Rosenquist Stiftung. Er war Mitglied eines wissenschaftlichen Beirats bei Family Larsson Rosenquist Foundation und der Task Force on Dietary Fat Quality der International Union of Nutritional Sciences. Sein Arbeitgeber erhielt Förderungen für wissenschaftliche Kooperationsprojekte von der Europäischen Kommission, dem European Research Council, der Deutschen Forschungsgemeinschaft, der Deutschen Bundesregierung, der Bayerischen Staatsregierung, den US National Institutes of Health, dem Ministry of Primary Industries New Zealand, der Else Kröner Fresenius Stiftung, der Familie Larsson Rosenquist Foundation, der Universität Amsterdam und den Unternehmen Bayer, Danone, DGC, Hipp, und Nestle. Sein Arbeitgeber hält Nutzungsrechte für zwei von BK angemeldete Patente zur Fettsäureanalyse und für eine Vorrichtung zur Gewinnung von Mundschleimhautzellproben.

B. Lawrenz übernimmt Berater-/Gutachtertätigkeiten mit Honorar für GSK, KVWL Consult GmbH, MSD, Pfizer und Sanofi. Auch arbeitet er in einem Beirat für GSK, MSD, Pfizer und Sanofi mit, von denen er Zuwendungen erhält. Er übernahm honorierte Vortrags-/Schulungstätigkeiten für den BVKJ, die BVKJ Service GmbH, GSK, KVWL Consult Life Science, MSD, RG und Sanofi. Er ist Autor/Koautor für das Hansische Verlagskontor sowie den Marseille-Verlag.

W. A. Mihatsch erhielt Zuwendungen für seine Tätigkeit im wissenschaftlichen Beirat für die Muttermilchforschung und des Ernährungspreis-Komitees der Firma Nutricia. Er übernahm Vortrags- und Schulungstätigkeiten für ÖGKJ, DGPE, GNPI, ESPGHAN, ESPR und DGKJ. Er führt Forschungsvorhaben/klinische Studien für die Uni Madrid durch und hält Vorträge zur Stillförderung.

C. Posovszky war Berater/Gutachter für die Nestlé Health Science GmbH Deutschland, von der er ein Honorar erhielt. Er arbeitete in wissenschaftlichen Beiräten der Shire Austria und der Shire Deutschland GmbH, Takeda (Schweiz), Nutricia Milupa und Pharmacosmos mit und erhielt hierfür Zuwendungen. CP erhielt Honorare für Vortrags-/Schulungstätigkeiten für AbbVie Deutschland GmbH, Nutricia GmbH, Nutricia Milupa SA, Takeda (Schweiz), Gebro Pharma GmbH Österreich, Publicare AG und FomF GmbH. Die Shire International GmbH sowie die FresuCare AG fördern Forschungsvorhaben von CP durch Drittmittel.

S. Rudloff erhielt Honorare für Vortrags-/Schulungstätigkeiten für die DGKJ, HIPP, Nutricia und Reisekostenerstattungen für das Heiner-Brunner-Seminar der GPGE.

Tabelle 1: Von der Europäischen Behörde für Lebensmittelsicherheit (EFSA) vorgeschlagene Höchstmengen (in g/l) für synthetische Oligosaccharide, die Oligosacchariden in der Frauenmilch strukturidentisch sind, bzw. ihrer Kombinationen in Säuglingsnahrungen.

**Oligosaccharide**	In Säuglingsanfangsnahrung	In Folgenahrung
2'FL	2,4	2,4
2'FL+DFL	1,6	1,2
LNnT	0,6	0,6
LNT	0,8	0,6
3'SL	0,2	0,15
6'SL	0,4	0,3

References

1. Bode L (2012) Human milk oligosaccharides: every baby needs a sugar mama. Glycobiology 22:1147–1162. https://doi.org/10.1093/glycob/cws074
2. Rudloff S, Kunz C (2015) Oligosaccharide in Frauenmilch. Monatsschr Kinderheilkd 163:790–795. https://doi.org/10.1007/s00112-014-3292-5
3. Asadpoor M, Peeters C, Henricks PAJ et al. (2020) Anti-Pathogenic Functions of Non-Digestible Oligosaccharides In Vitro. Nutrients 12. https://doi.org/10.3390/nu12061789
4. Prieto PA (2012) Profiles of human milk oligosaccharides and production of some human milk oligosaccharides in transgenic animals. Adv Nutr 3:456S-64S. https://doi.org/10.3945/an.111.001529
5. Urashima T, Hirabayashi J, Sato S et al. (2018) Human Milk Oligosaccharides as Essential Tools for Basic and Application Studies on Galectins. Trends in Glycoscience and Glycotechnology 30:SE51-SE65. https://doi.org/10.4052/tigg.1734.1SE
6. Lefebvre G, Shevlyakova M, Charpagne A et al. (2020) Time of Lactation and Maternal Fucosyltransferase Genetic Polymorphisms Determine the Variability in Human Milk Oligosaccharides. Front Nutr 7:574459. https://doi.org/10.3389/fnut.2020.574459
7. Kunz C, Meyer C, Collado MC et al. (2017) Influence of Gestational Age, Secretor, and Lewis Blood Group Status on the Oligosaccharide Content of Human Milk. J Pediatr Gastroenterol Nutr 64:789–798. https://doi.org/10.1097/MPG.0000000000001402
8. McGuire MK, Meehan CL, McGuire MA et al. (2017) What's normal? Oligosaccharide concentrations and profiles in milk produced by healthy women vary geographically. Am J Clin Nutr 105:1086–1100. https://doi.org/10.3945/ajcn.116.139980
9. Nordgren J, Sharma S, Bucardo F et al. (2014) Both Lewis and secretor status mediate susceptibility to rotavirus infections in a rotavirus genotype-dependent manner. Clinical infectious diseases: an official publication of the Infectious Diseases Society of America 59:1567–1573. https://doi.org/10.1093/cid/ciu633
10. Payne DC, Currier RL, Staat MA et al. (2015) Epidemiologic Association Between FUT2 Secretor Status and Severe Rotavirus Gastroenteritis in Children in the United States. JAMA Pediatr 169:1040–1045. https://doi.org/10.1001/jamapediatrics.2015.2002.
11. Ramani S, Stewart CJ, Laucirica DR et al. (2018) Human milk oligosaccharides, milk microbiome and infant gut microbiome modulate neonatal rotavirus infection. Nat Commun 9:5010. https://doi.org/10.1038/s41467-018-07476-4.
12. Andreas NJ, Al-Khalidi A, Jaiteh M et al. (2016) Role of human milk oligosaccharides in Group B Streptococcus colonisation. Clin Transl Immunology 5:e99. https://doi.org/10.1038/cti.2016.43.
13. Morrow AL, Ruiz-Palacios GM, Altaye M et al. (2004) Human milk oligosaccharides are associated with protection against diarrhea in breast-fed infants. J Pediatr 145:297–303. https://doi.org/10.1016/j.jpeds.2004.04.054
14. Stepans MBF, Wilhelm SL, Hertzog M et al. (2006) Early consumption of human milk oligosaccharides is inversely related to subsequent risk of respiratory and enteric disease in infants. Breastfeed Med 1:207–215. https://doi.org/10.1089/bfm.2006.1.207
15. Colston JM, Francois R, Pisanic N et al. (2019) Effects of Child and Maternal Histo-Blood Group Antigen Status on Symptomatic and Asymptomatic Enteric Infections in Early Childhood. J Infect Dis 220:151–162. https://doi.org/10.1093/infdis/jiz072

16. Muthumuni D, Miliku K, Wade KH et al. (2021) Enhanced Protection Against Diarrhea Among Breastfed Infants of Nonsecretor Mothers. Pediatr Infect Dis J 40:260–263. https://doi.org/10.1097/INF.0000000000003014
17. Sakanaka M, Gotoh A, Yoshida K et al. (2019) Varied Pathways of Infant Gut-Associated Bifidobacterium to Assimilate Human Milk Oligosaccharides: Prevalence of the Gene Set and Its Correlation with Bifidobacteria-Rich Microbiota Formation. Nutrients 12. https://doi.org/10.3390/nu12010071
18. Davis EC, Dinsmoor AM, Wang M et al. (2020) Microbiome Composition in Pediatric Populations from Birth to Adolescence: Impact of Diet and Prebiotic and Probiotic Interventions. Dig Dis Sci 65:706–722. https://doi.org/10.1007/s10620-020-06092-x
19. Ackerman DL, Doster RS, Weitkamp J-H et al. (2017) Human Milk Oligosaccharides Exhibit Antimicrobial and Antibiofilm Properties against Group B Streptococcus. ACS Infect Dis 3:595–605. https://doi.org/10.1021/acsinfecdis.7b00064
20. Cheng L, Akkerman R, Kong C et al. (2020) More than sugar in the milk: human milk oligosaccharides as essential bioactive molecules in breast milk and current insight in beneficial effects. Crit Rev Food Sci Nutr:1–17. https://doi.org/10.1080/10408398.2020.1754756.
21. Autran CA, Kellman BP, Kim JH et al. (2018) Human milk oligosaccharide composition predicts risk of necrotising enterocolitis in preterm infants. Gut 67:1064–1070. https://doi.org/10.1136/gutjnl-2016-312819
22. Wejryd E, Martí M, Marchini G et al. (2018) Low Diversity of Human Milk Oligosaccharides is Associated with Necrotising Enterocolitis in Extremely Low Birth Weight Infants. Nutrients 10. https://doi.org/10.3390/nu10101556
23. van Niekerk E, Autran CA, Nel DG et al. (2014) Human milk oligosaccharides differ between HIV-infected and HIV-uninfected mothers and are related to necrotizing enterocolitis incidence in their preterm very-low-birth-weight infants. J Nutr 144:1227–1233. https://doi.org/10.3945/jn.113.187799
24. Masi AC, Embleton ND, Lamb CA et al. (2020) Human milk oligosaccharide DSLNT and gut microbiome in preterm infants predicts necrotising enterocolitis. Gut. https://doi.org/10.1136/gutjnl-2020-322771
25. Quigley M, Embleton ND, McGuire W (2019) Formula versus donor breast milk for feeding preterm or low birth weight infants. Cochrane Database Syst Rev 7:CD002971. https://doi.org/10.1002/14651858.CD002971.pub5.
26. Goehring KC, Marriage BJ, Oliver JS et al. (2016) Similar to Those Who Are Breastfed, Infants Fed a Formula Containing 2'-Fucosyllactose Have Lower Inflammatory Cytokines in a Randomized Controlled Trial. J Nutr 146:2559–2566. https://doi.org/10.3945/jn.116.236919
27. Vazquez E, Barranco A, Ramirez M et al. (2016) Dietary 2'-Fucosyllactose Enhances Operant Conditioning and Long-Term Potentiation via Gut-Brain Communication through the Vagus Nerve in Rodents. PLoS One 11:e0166070. https://doi.org/10.1371/journal.pone.0166070
28. Fleming SA, Mudd AT, Hauser J et al. (2020) Human and Bovine Milk Oligosaccharides Elicit Improved Recognition Memory Concurrent With Alterations in Regional Brain Volumes and Hippocampal mRNA Expression. Front Neurosci 14:770. https://doi.org/10.3389/fnins.2020.00770
29. European-Commission (2016). "Commission Delegated Regulation (EU) 2016/127 of 25 September 2015 supplementing Regulation (EU) No 609/2013 of the European Parliament and of the Council as regards the specific compositional and information requirements for infant formula and follow-on formula and as regards requirements on information relating to infant and young child feeding." Official Journal of the European Union: L 25/21.

30. van Leeuwen SS, Te Poele EM, Chatziioannou AC et al. (2020) Goat Milk Oligosaccharides: Their Diversity, Quantity, and Functional Properties in Comparison to Human Milk Oligosaccharides. J Agric Food Chem 68:13469–13485. https://doi.org/10.1021/acs.jafc.0c03766
31. Barile D, Rastall RA (2013) Human milk and related oligosaccharides as prebiotics. Curr Opin Biotechnol 24:214–219. https://doi.org/10.1016/j.copbio.2013.01.008
32. Austin S, Bénet T (2018) Quantitative determination of non-lactose milk oligosaccharides. Anal Chim Acta 1010:86–96. https://doi.org/10.1016/j.aca.2017.12.036
33. Eussen SRBM, Mank M, Kottler R et al. (2021) Presence and Levels of Galactosyllactoses and Other Oligosaccharides in Human Milk and Their Variation during Lactation and According to Maternal Phenotype. Nutrients 13. https://doi.org/10.3390/nu13072324
34. EFSA-Panel-on-Dietetic-Products (2014) Scientific Opinion on the essential composition of infant and follow-on formulae. EFS2 12:3760. https://doi.org/10.2903/j.efsa.2014.3760
35. Skórka A, Pieścik-Lech M, Kołodziej M et al. (2018) Infant formulae supplemented with prebiotics: Are they better than unsupplemented formulae? An updated systematic review. Br J Nutr 119:810–825. https://doi.org/10.1017/S0007114518000120
36. EFSA (2011) Scientific Opinion on the substantiation of health claims related to fructo-oligosaccharides (FOS) and decreasing potentially pathogenic gastro-intestinal microorganisms (ID 781) pursuant to Article 13(1) of Regulation (EC) No 1924/2006. EFSA Journal 9:2222
37. EFSA (2011) Scientific Opinion on the substantiation of health claims related to galacto oligosaccharides (GOS) and reduction of gastro intestinal discomfort (ID 763) and decreasing potentially pathogenic microorganisms (ID 765) pursuant to Article 13(1) of Regulation (EC) No 1924/2006. EFSA Journal 9:2060
38. Bode L, Contractor N, Barile D et al. (2016) Overcoming the limited availability of human milk oligosaccharides: challenges and opportunities for research and application. Nutr Rev 74:635–644. https://doi.org/10.1093/nutrit/nuw025
39. Sprenger GA, Baumgärtner F, Albermann C (2017) Production of human milk oligosaccharides by enzymatic and whole-cell microbial biotransformations. J Biotechnol 258:79–91. https://doi.org/10.1016/j.jbiotec.2017.07.030
40. EFSA (2015) Safety of 2′-O-fucosyllactose as a novel food ingredient pursuant to Regulation (EC) No 258/97. EFS2 13. https://doi.org/10.2903/j.efsa.2015.4184
41. EFSA (2015) Safety of lacto-N-neotetraose as a novel food ingredient pursuant to Regulation (EC) No 258/97. EFS2 13. https://doi.org/10.2903/j.efsa.2015.4183
42. Turck D, Castenmiller J, Henauw S de et al. (2019) Safety of 2'-fucosyllactose/difucosyllactose mixture as a novel food pursuant to Regulation (EU) 2015/2283. EFS2 17:e05717. https://doi.org/10.2903/j.efsa.2019.5717
43. Turck D, Castenmiller J, Henauw S de et al. (2019) Safety of lacto-N-tetraose (LNT) as a novel food pursuant to Regulation (EU) 2015/2283. EFS2 17:e05907. https://doi.org/10.2903/j.efsa.2019.5907
44. Turck D, Castenmiller J, Henauw S de et al. (2020) Safety of 3′-Sialyllactose (3′-SL) sodium salt as a novel food pursuant to Regulation (EU) 2015/2283. EFS2 18. https://doi.org/10.2903/j.efsa.2020.6098
45. Turck D, Castenmiller J, Henauw S de et al. (2020) Safety of 6′-Sialyllactose (6′-SL) sodium salt as a novel food pursuant to Regulation (EU) 2015/2283. EFS2 18. https://doi.org/10.2903/j.efsa.2020.6097
46. Marriage BJ, Buck RH, Goehring KC et al. (2015) Infants Fed a Lower Calorie Formula With 2'FL Show Growth and 2'FL Uptake Like Breast-Fed Infants. J Pediatr Gastroenterol Nutr 61:649–658. https://doi.org/10.1097/MPG.0000000000000889

47. Puccio G, Alliet P, Cajozzo C et al. (2017) Effects of Infant Formula With Human Milk Oligosaccharides on Growth and Morbidity: A Randomized Multicenter Trial. J Pediatr Gastroenterol Nutr 64:624–631. https://doi.org/10.1097/MPG.0000000000001520
48. Berger B, Porta N, Foata F et al. (2020) Linking Human Milk Oligosaccharides, Infant Fecal Community Types, and Later Risk To Require Antibiotics. mBio 11. https://doi.org/10.1128/mBio.03196-19
49. Storm HM, Shepard J, Czerkies LM et al. (2019) 2'-Fucosyllactose Is Well Tolerated in a 100% Whey, Partially Hydrolyzed Infant Formula With Bifidobacterium lactis: A Randomized Controlled Trial. Glob Pediatr Health 6:2333794X19833995. https://doi.org/10.1177/2333794X19833995
50. Larsson MW, Lind MV, Laursen RP et al. (2019) Human Milk Oligosaccharide Composition Is Associated With Excessive Weight Gain During Exclusive Breastfeeding-An Explorative Study. Front Pediatr 7:297. https://doi.org/10.3389/fped.2019.00297
51. Bode L (2019) Human Milk Oligosaccharides: Next-Generation Functions and Questions. Nestle Nutr Inst Workshop Ser 90:191–201. https://doi.org/10.1159/000490306.
52. Europäische Kommission (1999) "RICHTLINIE 1999/21/EG DER KOMMISSION vom 25. März 1999 über diätetische Lebensmittel für besondere medizinische Zwecke.". Amtsblatt der Europäischen Kommission L 091: 29-36.
53. Bundesministerium der Justiz und für Verbraucherschutz (2017) Verordnung über diätetische Lebensmittel (Diätverordnung). https://www.gesetze-im-internet.de/di_tv/BJNR004150963.html.
